# 6-Shogaol Antagonizes the Adipocyte-Conditioned Medium-Initiated 5-Fluorouracil Resistance in Human Colorectal Cancer Cells through Controlling the SREBP-1 Level

**DOI:** 10.3390/life11101067

**Published:** 2021-10-10

**Authors:** Ko-Chao Lee, Kuen-Lin Wu, Chia-Kung Yen, Cheng-Nan Chen, Shun-Fu Chang, Wen-Shih Huang

**Affiliations:** 1 Department of Colorectal Surgery, Department of Surgery, Kaohsiung Chang Gung Memorial Hospital, Kaohsiung Medical Center, Kaohsiung 833, Taiwan; kmch4329@gmail.com (K.-C.L.); focus913@gmail.com (K.-L.W.); 2 Department of Food Science, National Chiayi University, Chiayi 600, Taiwan; Maple313@gmail.com; 3 Department of Biochemical Science and Technology, National Chiayi University, Chiayi 600, Taiwan; cnchen@mail.ncyu.edu.tw; 4 Department of Medical Research and Development, Chiayi Chang Gung Memorial Hospital, Chiayi 613, Taiwan; sfchang@cgmh.org.tw; 5 Graduate Institute of Clinical Medical Sciences, College of Medicine, Chang Gung University, Taoyuan 333, Taiwan; 6 Division of Colon and Rectal Surgery, Department of Surgery, Chiayi Chang Gung Memorial Hospital, Chiayi 613, Taiwan

**Keywords:** adipocyte-conditioned medium, AMPK, colorectal cancer, 5-fluorouracil, SREBP-1

## Abstract

The resistance of colorectal cancer (CRC) to chemotherapy, e.g., 5-fluorouracil (5-FU), is an impediment to successful cancer treatment. Although many mechanisms have been proposed to explain the occurrence of resistance, little is known concerning the role of the adipocyte-containing microenvironment of CRC. Accumulating data have proposed that the combined therapy of clinical drugs with ginger derivatives, e.g., 6-shogaol, might improve resistance development. In the present study, we examined the effect of adipocyte-conditioned medium (ACM) on 5-FU-treated CRC cells (human DLD-1 and SW480 cells) and further examined the possible antagonized role of 6-shogaol in this situation. It was shown that the level of sterol-regulatory element-binding protein-1 (SREBP-1), a critical transcription factor involved in lipid synthesis and metabolism, would be upregulated through Akt and p70S6K signaling pathways while CRC cells are cultured in ACM, which subsequently decreases the cell sensitivity to 5-FU cytotoxicity. Moreover, our results also demonstrated the antagonized role of 6-shogaol in attenuating the ACM effects on CRC cells through activating AMPK signaling. Overall, the present study elucidated the role of adipocyte-containing microenvironment in 5-FU resistance development of CRC through controlling the SREBP-1 level and further enhanced the concept of clinical application of 6-shogaol and AMPK signaling in CRC therapy.

## 1. Introduction

Colorectal cancer (CRC) still accounts for the most common cancer types and cancer-associated deaths in the world [1,2]. Although the CRC screening has been promoted and conducted in many countries for more than 10 years, the incidence is still elevated annually. Moreover, the rate of CRC patients whose age is below 50 years also increased rapidly in the past decade [1,2]. It has long been indicated that a poor diet with excess calorie intake is still the major cause of CRC development. Moreover, in spite of the improvement and advance of the therapeutic strategy, including chemotherapy and radiation, one of the most challenges of CRC has also been the occurrence of resistance [3]. Thus, a more detailed and precise study of resistance development about biomarker screen and the underlying mechanism has also been thought to be necessary and urgent.

Obesity has long been indicated as one of the risk factors for CRC. An individual with obesity would increase their fat mass and inflammatory adipose tissue phenotype, which subsequently results in the progression of chronic inflammation status in the body [4]. This situation has been directly associated with the major cause of CRC development. Adipocytes within the adipose tissue have been shown not just a simply energy-producing resource and storehouse but also contribute to the secretion of various paracrine and endocrine factors, including cytokines and adipokines, to influence cell function and inflammation [5,6]. Recently, accumulating clinical evidence has found that CRC patients with obesity might have more difficulty in their therapeutic efficacy after chemotherapy [5,6]. Because of the presence and their proximity to cancer tissue, it has been proposed that adipocytes might affect the phenotype and behavior of cancer cells, including abnormal growth and metastasis, through their secretory factors [7]. Moreover, this situation has also been considered as a cause of resistance development of chemotherapy and radiotherapy, but the detailed mechanism needs more information [8].

Abnormal lipid metabolism found in most cancer types has been implicated in carcinogenesis and further malignant development [9,10,11]. Sterol-regulatory element-binding protein-1 (SREBP-1) is a critical transcription factor involved in regulating the expressions of many enzymes in lipid synthesis and metabolic process. SREBP-1 has been indicated as a cholesterol sensor, which is located in the endoplasmic reticulum to regulate intracellular cholesterol homeostasis [12]. Under the stimulation of insulin signaling, pro-SREBP-1 would be transported from the endoplasmic reticulum to the Golgi to be further processed by proteases. After that, SREBP1 could be activated and further translocated to the nucleus to control the gene expression [13]. Recently, in addition to the regulatory role of lipid homeostasis, accumulating data have found that abnormal SREBP-1 expression levels might affect the development of cancers, including ovarian, breast, and CRC, and initiate the occurrence of therapeutic resistance [14]. However, the precise role in these correlations has not been elucidated clearly.

Because of the inevitableness of adverse effect and drug resistance for chemotherapy, the nature herbaceous plants, e.g., ginger, has been extensively investigated and shown their fantastic applications in cancer treatment [15,16,17,18]. 6-shogaol, one of the major bioactive components of ginger, has been found its potential role in anti-oxidation anti-inflammation. Moreover, in a cancer study, 6-shogaol has been indicated as a great cancer cell killer to many types of cancers [15,16,17,18]. Moreover, accumulating evidence has also found that 6-shogaol could effectively improve the development of chemotherapy resistance [17,18]. Because the development of cancer is an extremely complicated process, currently, chemical drugs are still irreplaceable for treating patients with cancers. However, the combined therapy with chemical drugs and natural herbaceous plants might be a great strategy for attenuating the patients’ uncomfortable and drug resistance development. Hence, 6-shogaol could be a potential candidate and should be further examined in detail.

In the present study, we aimed to determine if the chemotherapy efficiency of CRC cells with 5-fluorouracil (5-FU) could be influenced while cells were cultured in an adipocyte-conditioned medium (ACM). We also further examined the possible improved role of 6-shogaol in this resistance-initiating process. It was shown that ACM activates Akt and p70S6K signaling pathways to regulate the SREBP-1 expression and hence decreased the cell sensitivity to 5-FU cytotoxicity in both DLD-1 and SW480 CRC cells. Moreover, 6-shogaol could effectively attenuate this resistance development through activating the AMPK signaling.

## 2. Materials and Methods

### 2.1. Materials

The culture materials (medium/antibiotics/FBS) were purchased from Gibco (Grand Island, NY, USA). Chemical inhibitors for protein kinases, including LY294002 for Akt and rapamycin for p70S6K, were purchased from Sigma (St. Louis, MO, USA). Specific antibodies against SREBP-1, phosphor-Akt, Akt, phosphor-p70S6K, p70S6K, and β-actin were purchased from Cell Signaling Technology (Beverly, MA, USA). The control-, SREBP-1- and AMPK-specific siRNAs were purchased from Thermo (Waltham, MA, USA). All other chemicals were obtained from Sigma (St. Louis, MO, USA).

### 2.2. Cell Culture

Human DLD-1 and SW480 CRC cells from the cell bank in the Taiwan Food Industry Research and Development Institute (Hsinchu, Taiwan) were cultured and maintained in commercial Dulbecco’s Modified Eagle Medium (DMEM) with 10% FBS and 1% penicillin/streptomycin in a culture incubator with 5% CO_2_ and 37 °C temperature.

### 2.3. MTT Assay

Human DLD-1 and SW480 CRC cells were cultured in a culture incubator with 5% CO_2_ and 37 °C temperature overnight, and then their cell viability was examined by 3-(4,5-dimethylthiazol-2-yl)-2,5-diphenyltetrazolium bromide (MTT) assay. The treated cells were further treated with MTT reagent (0.5 mg/mL) and incubated for another 3 h in a culture incubator with 5% CO_2_ and 37 °C temperature. After that, DMSO was added to the wells to dissolve the formazan crystals, and then the absorbance (570 nm) was examined.

### 2.4. Real-Time PCR

The total RNAs of treated cells were isolated and purified by using the extraction kit (Trizol reagent). After quantification, the equal concentration of RNA was converted to cDNA by using the reverse-transcription kit. Real-time PCR assay was conducted with the SYBR Green system (Thermo, Waltham, MA, USA) in a Bio-Rad machine (Hercules, CA, USA). The specific primers for examined genes included (i) SREBP-1 (positive: 5′-CAGGTACCGAGTTCTGGTGTGTTGGGCCA-3′; negative: 5′-ACTGCTAGCCGCGCTGCCGCCTCGCTAG-3′) and (ii) GAPDH (positive: 5′-AGG TGAAGGTCGGAGTCAAC-3′; negative: 5′-CCATGTAGTTGAGGTCAATGAAGG-3′). GAPDH level was defined as the internal control.

### 2.5. Western Blot

After treatment, the human DLD-1 and SW480 CRC cells were lysed by adding the commercial lysis buffer and a protease/phosphatase inhibitor cocktail (Figure S1). The protein lysates were quantified by using the protein quantification kit (Bio-Rad, Hercules, CA, USA). Equal concentrations of proteins (30 µg) were loaded into and separated in the SDS-PAGE with 4% stacking up-gel and 10% running down-gel. After that, the separated proteins were further transferred onto the nitrocellular paper and then detected and analyzed by incubated with the indicated antibodies.

### 2.6. DN-Akt and siRNA Transfection

Human DLD-1 and SW480 CRC cells were cultured and maintained in a commercial DMEM medium with 10% FBS in a culture incubator with 5% CO_2_ and 37 °C temperature overnight and then transfected with SREBP-1-specific siRNA, AMPK-specific siRNA, or DN-Akt by using the lipofectamine 2000 transfection kit (Thermo, Waltham, MA, USA). After further 48 h incubation, the cells were used in the other assay.

### 2.7. Statistical Analysis

The statistical data were presented by mean ± standard error of the mean. The data were measured by an independent Student *t*-test for two groups of data and analysis of variance (ANOVA) followed by Scheffe’s test for multiple comparisons. A value lower than 0.05 was defined as significant.

## 3. Results

**Figure 1:**
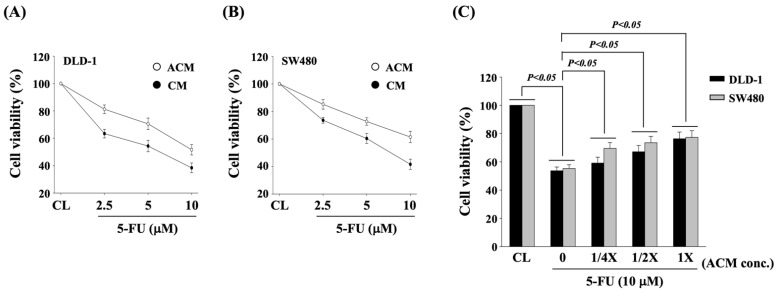
ACM decreases the cytotoxic effect of 5-FU on DLD-1 and SW480 CRC cells. (**A**,**B**) DLD-1 and SW480 CRC cells were cultured in control medium (CM) or adipocyte-conditional medium (ACM) for 8 h and then were kept as controls or treated with 5-FU (2.5, 5, and 10 μM) for 24 h. (**C**) DLD-1 and SW480 CRC cells were cultured in CM to serve as controls (CL) or cultured in ACM with different concentrations for 8 h and then were further kept as controls (0) or treated with 5-FU (10 μM) for 24 h. (**A**–**C**) The cell viability was assayed by the MTT assay. Data are shown as mean ± SEM from three independent experiments. *p* < 0.05 was defined as significant.

ACM decreases the cytotoxic effect of 5-FU on DLD-1 and SW480 CRC cells. DLD-1 and SW480 CRC cells were cultured in control medium (CM) or adipocyte-conditional medium (ACM) for 8 h and then were kept as controls or treated with 5-FU (2.5, 5, and 10 μM) for 24 h. The cell viability of treated cells was examined by MTT assay. It was shown that 5-FU dose-dependently induces the cell death of both types of CRC cells cultured in both CM and ACM (Figure 1A). However, cells cultured in ACM showed less sensitivity to 5-FU cytotoxic effect (Figure 1A,B). Moreover, cells cultured in ACM with different concentrations further showed a dose-dependent antagonized effect to 5-FU cytotoxicity in both DLD-1 and SW480 CRC cells (Figure 1C).

**Figure 2:**
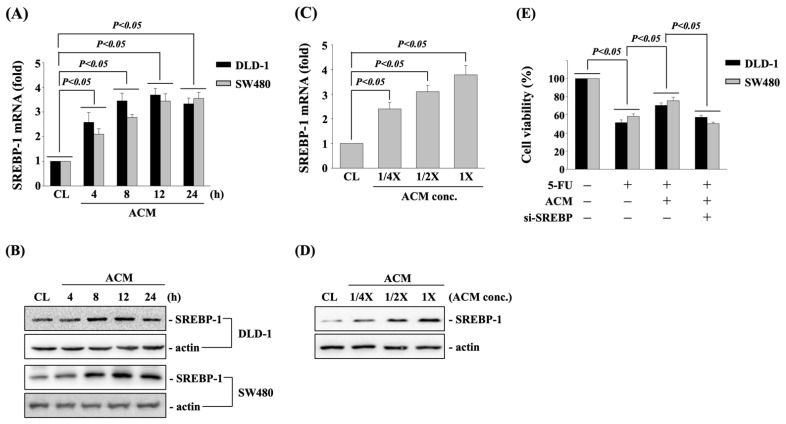
ACM upregulates the SREBP-1 to affects the cytotoxicity of 5-FU in DLD-1 and SW480 CRC cells. **(A**–**D**) DLD-1 and SW480 CRC cells were cultured in CM to serve as controls (CL) or (**A**,**B**) cultured in ACM for 4, 8, 12, and 24 h or (**C**,**D**) cultured in ACM with different concentrations for 8 h and then the (**A**,**C**) mRNA and (**B**,**D**) protein expressions of SREBP-1 were examined by real-time PCR and western blot, respectively. (**E**) DLD-1 and SW480 CRC cells were transfected with SREBP-1-specific siRNA and then cultured in CM or ACM for 8 h. After that, cells were kept as controls or treated with 5-FU (10 μM) for 24 h, and the cell viability was examined by MTT assay. Data in (**A**,**C**,**E**) are shown as mean ± SEM from three independent experiments. Results in (**B**,**D**) are representative of three independent experiments with similar results. *p* < 0.05 was defined as significant.

ACM upregulates the SREBP-1 to affects the cytotoxicity of 5-FU in DLD-1 and SW480 CRC cells. DLD-1 and SW480 CRC cells were cultured in CM to serve as controls or cultured in ACM for 4, 8, 12, and 24 h, and then the mRNA and protein expressions of SREBP-1 were examined. Cultivation of cells with ACM significantly induces SREBP-1 mRNA (Figure 2A) and protein (Figure 2B) expressions in both DLD-1 and SW480 CRC cells within 4 h and 8 h, respectively, and then slightly declines after 24 h treatment. Moreover, DLD-1 CRC cells treated with different doses of ACM for 8 h further showed that ACM induces the expressions of SREBP-1 mRNA (Figure 2C) and protein (Figure 2D) in the dose-dependent manners compared to the control cells. To determine the role of SREBP-1 upregulation in ACM-antagonized 5-FU cytotoxicity in both types of CRC cells, DLD-1 and SW480 CRC cells were transfected with SREBP-1-specific siRNA and then cultured in CM or ACM for 8 h. After that, cells were kept as controls or treated with 5-FU (10 μM) for 24 h, and the cell viability was examined by MTT assay. It was shown that the knockdown of SREBP-1 gene expression decreased the antagonized effect of ACM on 5-FU-induced cell death of both types of CRC cells (Figure 2E).

**Figure 3:**
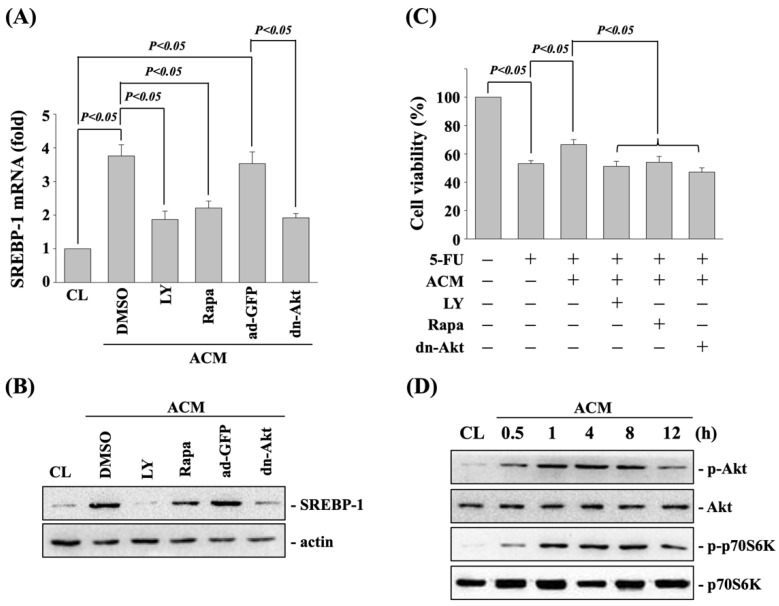
Akt and p70S6K signaling pathways regulate the ACM-affected SREBP-1 upregulation and 5-FU cytotoxicity in DLD-1 CRC cells. (**A**–**C**) DLD-1 CRC cells were cultured in CM to serve as controls (CL) or pretreated with DMSO or LY294002 (Akt inhibitor, 20 μM), or rapamycin (p70S6k inhibitor, 100 nM) for 1 h or pretreated with ad-GFP- or dn-Akt-expressed plasmids for 48 h and then cultured in ACM for 8 h to analyze the (**A**) SREBP-1 mRNA (real-time PCR) and (**B**) protein (western blot) expressions or cultured in ACM for 8 h with 5-FU for 24 h to analyze the (**C**) cell viability (MTT assay). (**D**) DLD-1 CRC cells were cultured in CM to serve as controls (CL) or cultured in ACM for 0.5, 1, 4, 8, and 12 h, and then the phosphorylations and expressions of Akt and p70S6K proteins were examined by western blot. Data in (**A**,**C**) are shown as mean ± SEM from three independent experiments. Results in (**B**,**D**) are representative of three independent experiments with similar results. *p* < 0.05 was defined as significant.

Akt and p70S6K signaling pathways regulate the ACM-affected SREBP-1 upregulation and 5-FU cytotoxicity in DLD-1 CRC cells. DLD-1 CRC cells were cultured in CM to serve as controls or pretreated with DMSO or LY294002 (Akt inhibitor, 20 μM), or rapamycin (p70S6k inhibitor, 100 nM) for 1 h or pretreated with ad-GFP- or dn-Akt-expressed plasmids for 48 h and then cultured in ACM for 8 h to analyze the SREBP-1 mRNA (real-time PCR) and protein (western blot) expressions or cultured in ACM for 8 h with 5-FU for 24 h to analyze the cell viability (MTT assay). It was shown that the activity inhibition of Akt and p70S6K kinases in DLD-1 CRC cells significantly attenuate the ACM-increased SREBP-1 mRNA (Figure 3A) and protein (Figure 3B) expressions and ACM-antagonized 5-FU cytotoxicity (Figure 3C) compared to the DMSO- or ad-GFP-pretreated cells. Moreover, cells treated with ACM for 0.5, 1, 4, 8, and 12 h induced phosphorylations of Akt and p70S6k kinases in DLD-1 CRC cells within 0.5 h and persisted for 12 h after treatment (Figure 3D).

**Figure 4:**
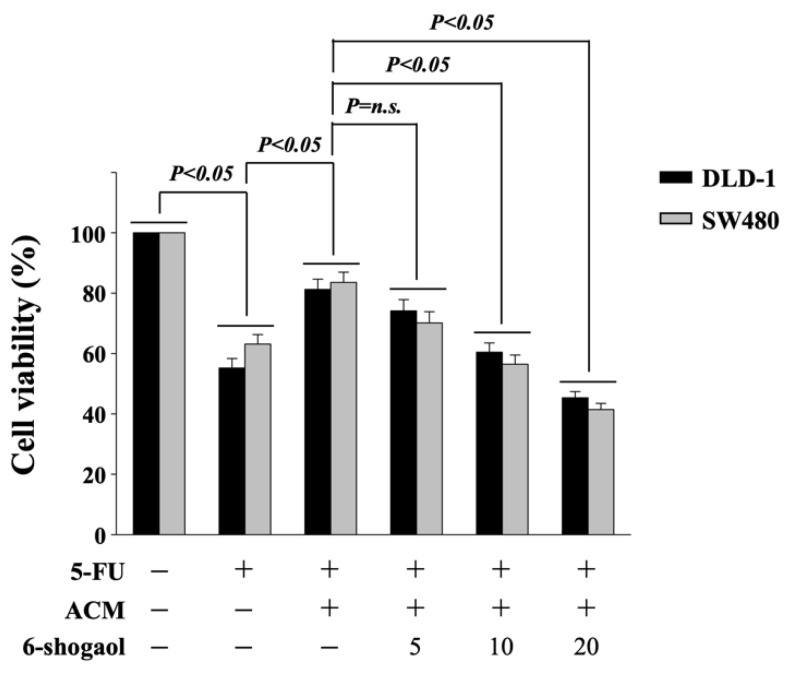
6-shogaol recovers the ACM effect on CRC sensitivity to 5-FU cytotoxicity in both DLD-1 and SW480 CRC cells. DLD-1 and SW480 CRC cells were cultured in CM to serve as controls or pretreated with 6-shogaol (5, 10, 20 μM) for 1 h and then cultured in ACM for 8 h with 5-FU for 24 h to analyze the cell viability (MTT assay). Data are shown as mean ± SEM from three independent experiments. *p* < 0.05 was defined as significant. n.s. is the abbreviation of no significance.

**Figure 5:**
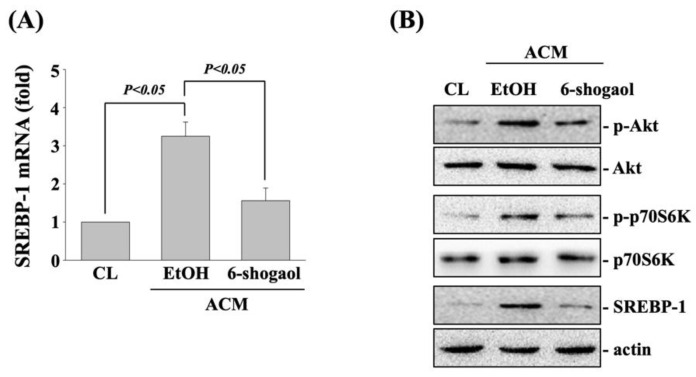
6-shogaol recovers the ACM effects on SREBP-1 upregulation and Akt/p70S6K phosphorylation in DLD-1 CRC cells. (**A**,**B**) DLD-1 CRC cells were cultured in CM to serve as controls (CL) or pretreated with ethanol (EtOH) or 6-shogaol (20 μM) for 1 h and then cultured in ACM for 8 h. After that, (**A**,**B**) the mRNA (real-time PCR) and protein (western blot) expression of SREBP-1 and (**B**) the phosphorylations and expressions of Akt and p70S6K proteins (western blot) were examined. Data in (**A**) are shown as mean ± SEM from three independent experiments. Results in (**B**) are representative of three independent experiments with similar results. *p* < 0.05 was defined as significant.

6-shogaol recovers the ACM effects on 5-FU-induced cell death, SREBP-1 upregulation, and Akt/p70S6K phosphorylation in CRC cells. 6-shogaol has been indicated to be a great natural product for anticancer [15,16,17,18]. Next, we determined if 6-shogaol influences the ACM effect on both types of CRC cells. DLD-1 and SW480 CRC cells were cultured in CM to serve as controls or pretreated with 6-shogaol (5, 10, 20 μM) for 1 h and then cultured in ACM for 8 h with 5-FU for 24 h to analyze the cell viability (MTT assay). It was shown that cells treated with 10 and 20 μM 6-shogaol significantly attenuated the ACM-antagonized 5-FU cytotoxicity in both DLD-1 and SW480 CRC cells (Figure 4). Moreover, DLD-1 CRC cells treated with 20 μM 6-shogaol also decreased the ACM-induced expressions of SREBP-1 mRNA (Figure 5A) and protein (Figure 5B) and phosphorylations of Akt and p70S6k kinases (Figure 5B).

**Figure 6:**
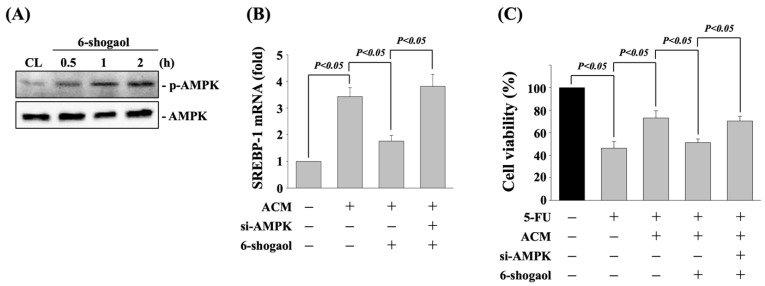
6-shogaol activates AMPK signaling pathway to attenuate the ACM effect on SREBP-1 expression and 5-FU-induced cell death in DLD-1 CRC cells. (**A**) DLD-1 CRC cells cultured in CM to serve as controls (CL) or treated with 6-shogaol (20 μM) for 0.5, 1, and 2 h, and then the AMPK phosphorylation was analyzed by western blot. (**B**,**C**) DLD-1 CRC cells were cultured in CM to serve as controls or transfected with AMPK-specific siRNA to knock down the respective gene expression for 48 h and then cultured in ACM or ACM + 6-shogaol. After that, (**B**) the SREBP-1 mRNA expression was analyzed by real-time PCR, and (**C**) the cell viability was analyzed by MTT assay. Results in (**A**) are representative of three independent experiments with similar results. Data in (**B**,**C**) are shown as mean ± SEM from three independent experiments. *p* < 0.05 was defined as significant.

6-shogaol activates the AMPK signaling pathway to attenuate the ACM effect on SREBP-1 expression and 5-FU-induced cell death in DLD-1 CRC cells. DLD-1 CRC cells were cultured in CM to serve as controls (CL) or treated with 6-shogaol (20 μM) for 0.5, 1, and 2 h, and then the AMPK phosphorylation was analyzed by western blot. It was shown that 6-shogaol time-dependently induces AMPK phosphorylation within 0.5 h and persisted for 2 h after treatment (Figure 6A). Moreover, cells treated with AMPK-specific siRNA to knock down the respective gene expression significantly recovered the 6-shogaol effect on decreasing the ACM-induced SREBP-1 mRNA expression (Figure 6B) and the ACM-antagonized 5-FU cytotoxicity (Figure 6C).

## 4. Discussion

Our systematic experiments demonstrated that (i) DLD-1 and SW480 CRC cells cultured in ACM environment could upregulate the SREBP-1 expression and hence decrease their sensitivity to 5-FU cytotoxicity; (ii) Akt and p70S6K signaling pathways regulate the ACM effect on SREBP-1 upregulation and 5-FU-induced cell death in CRC cells. (iii) 6-shogaol could attenuate these ACM effects on both DLD-1 and SW480 CRC cells through activating the AMPK signaling pathway. This study revealed the role of SREBP-1 upregulation in initiating 5-FU resistance in CRC cells in response to the ACM environment and elucidated the underlying mechanism. Moreover, this study also found that the combined therapy of 5-FU with 6-shogaol could adequately improve the drug resistance development of CRC cells to 5-FU cytotoxicity

SREBP-1 is a nuclear protein that could act as a transcription factor involved in cholesterol metabolism and the modulation of lipid synthesis-related gene transcription. However, increasing evidence has also supported the concept that SREBP-1 could play an important role in tumor progression and malignancy [19,20,21,22]. Moreover, a positive correlation between CRC development and SREBP-1 upregulation has been further found in clinical findings [22,23]. This clinical evidence has indicated that (i) CRC tissues from patients show a more elevated SREBP-1 level than noncancerous tissues [22] and (ii) SREBP-1 mRNA level is higher in colon carcinomas [23]. Lipid is one of the energy resources for normal cell survival and is also a major ingredient of cell membranes. Therefore, an explanation of the clinical positive correlation between SREBP-1 upregulation and cancer development is that SREBP-1 increases the lipid synthesis in order to flatter the abnormal energy demand and cell division of cancer cells [19]. On the other hand, SREBP-1 has also been reported to affect the apoptosis of cancer cells by inhibiting the expression of the apoptotic factor [24]. Moreover, recent studies have further revealed that SREBP-1 might initiate the development of drug resistance in hepatocellular carcinoma and CRC [25,26,27]. All of these above data support our findings that ACM treatment could induce SREBP-1 mRNA and protein expressions and subsequently reduce the sensitivity of 5-FU cytotoxicity in both human DLD-1 and SW480 CRC cells. Moreover, these studies, including ours, also support that SREBP-1 could be an important target of the theranostic tool and drug development for CRC patients.

6-shogaol has been indicated as a great candidate of natural herbs for anticancer therapy [15,16]. Tumorigenesis is an extremely complex process that involves abnormal signaling perturbations and various gene mutations. Moreover, this process is also associated with the occurrence of chronic inflammation. Hence, because of the anti-oxidation and anti-inflammation properties, 6-shogaol has been examined and demonstrated its efficacy in treating the various types of cancers [28,29]. Our data further found that 6-shogaol could effectively decrease the SREBP-1 upregulation, which was induced because CRC cells were in the ACM environment. Moreover, this effect would further increase the sensitivity of CRC cells to 5-FU cytotoxicity. In addition to the cancer-killing capability, accumulating data, including ours, has also found the 6-shogaol effect on attenuating the development of drug resistance [17,18]. It has been suggested that the main cause and mechanism of 6-shogaol efficacy is to activate the AMPK signaling, which is responsible for the re-regulation of energy homeostasis in cancer cells. According to the extensive study outcomes, 6-shogaol has already been confirmed its low toxic and low side-effect in healthy tissues. Hence, combined with these findings, it could be solidly suggested that 6-shogaol could be effectively applied in the clinical combined therapy strategy to attenuate the side-effect and resistance of chemotherapy.

AMPK, an intracellular energy sensor and controller, has been originally served as a therapeutic target for the treatment of diabetes [30]. However, the regulation of AMPK signaling has also been further reported to potentiate the cytotoxicity and efficacy of anticancer drugs in treating patients with different types of cancers [31,32]. Moreover, this is also because of its energy homeostasis regulatory mechanism [30]. It has been shown that (i) AMPK activation by adding the AICAR, an agonist, significantly increasing the efficacious treatment for lethal breast cancer [31,32]; (ii) AMPK activation could promote the mitochondrial activity to increase the treating sensitivity in patients with myeloid leukemia [33]; (iii) AICAR activates the AMPK signaling to enhance the anticancer effect of chemotherapeutic agents in prostate cancer cells [34]. These data showing the better cancer-killing efficacy through AMPK activation support our present results that AMPK activation induced by 6-shogaol could attenuate the SREBP-1 expression and hence enhance the 5-FU-induced cytotoxicity of ACM-treated CRC cells.

## 5. Conclusions

Our results found that SREBP-1 could be upregulated while the CRC cells are under the ACM environment, and this effect would further decrease the sensitivity of CRC cells to 5-FU cytotoxicity. Moreover, the combined therapy of 5-FU with 6-shogaol could activate the AMPK signaling to recover the effect of SREBP-1 upregulation. Overall, the present study elucidated the relationship between the adipocyte-containing microenvironment and 5-FU resistance development of CRC cells through regulating the SREBP-1 expression level and further enhanced the concept of clinical application of 6-shogaol and AMPK signaling in CRC therapy.

## Data Availability

The original images of Western blot results (Figure 2, Figure 3, Figure 5 and Figure 6) are presented in Supplementary Materials Figure S1.

## Supplementary Materials

The following are available online at https://www.mdpi.com/article/10.3390/life11101067/s1, Figure S1: Gel images of Western blot results (Figure 2, Figure 3, Figure 5 and Figure 6).
