# Supplementary material for: 6-Shogaol Antagonizes the Adipocyte-Conditioned Medium-Initiated 5-Fluorouracil Resistance in Human Colorectal Cancer Cells through Controlling the SREBP-1 Level
Source: Life (Basel). 2021 Oct 10;11(10):1067. doi: 10.3390/life11101067 (PMC8537026; doi:10.3390/life11101067)
Supplement: Supplementary file 1 [file life-11-01067-s001.zip › Figure S1 Raw data for life-1387314-Revised 1.pdf]

Figure 2B

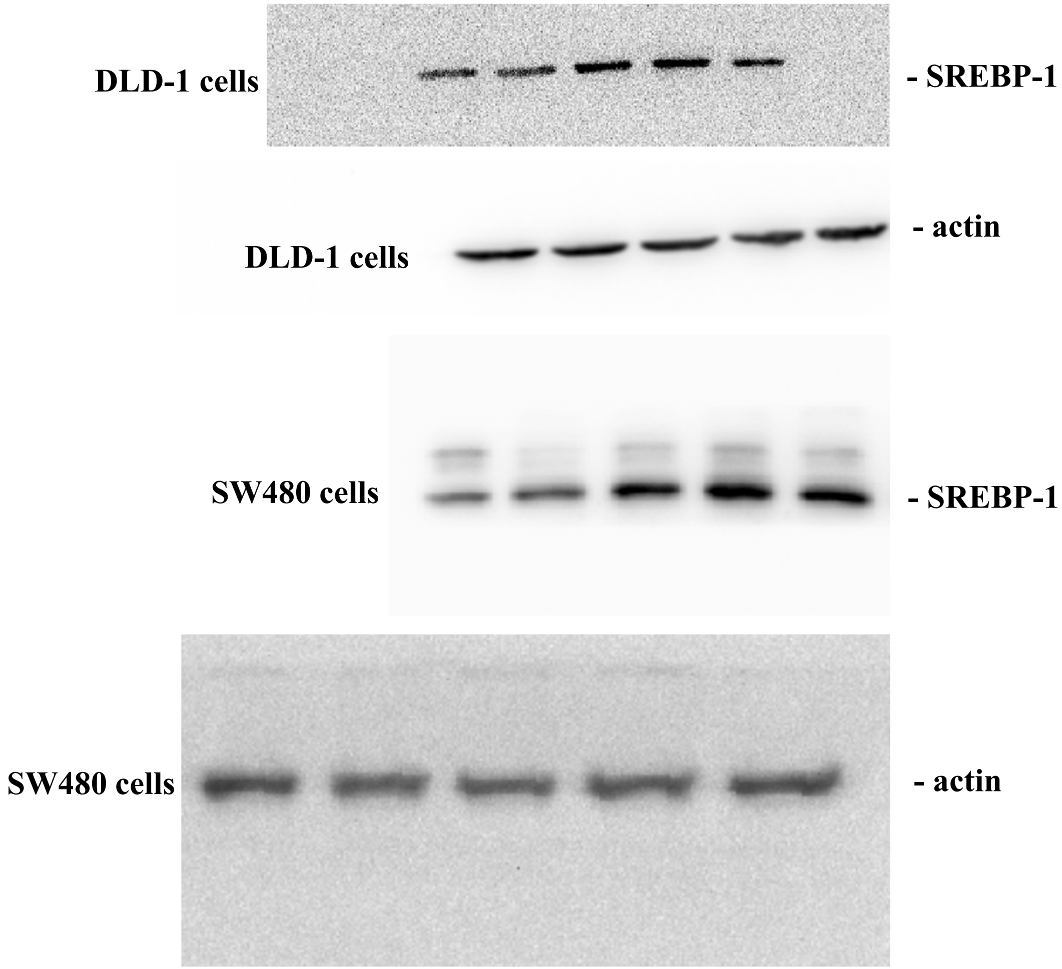

Figure 2D

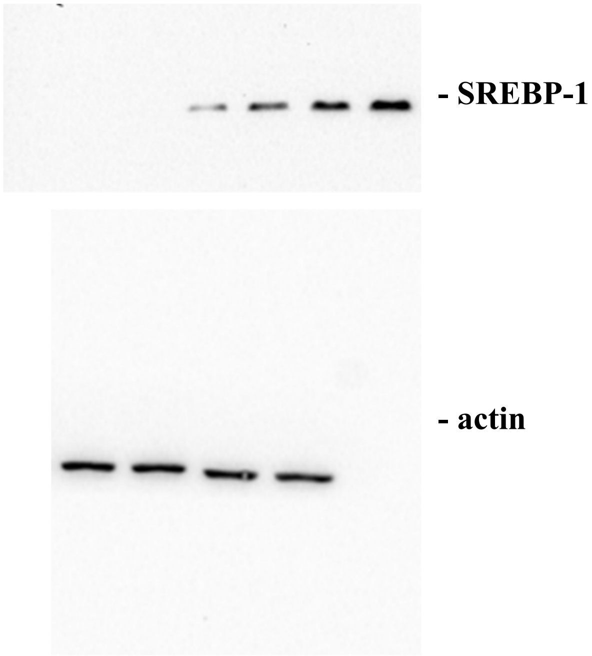

Figure S1: Gel images of Western blot in Figure 2.

Figure 3B

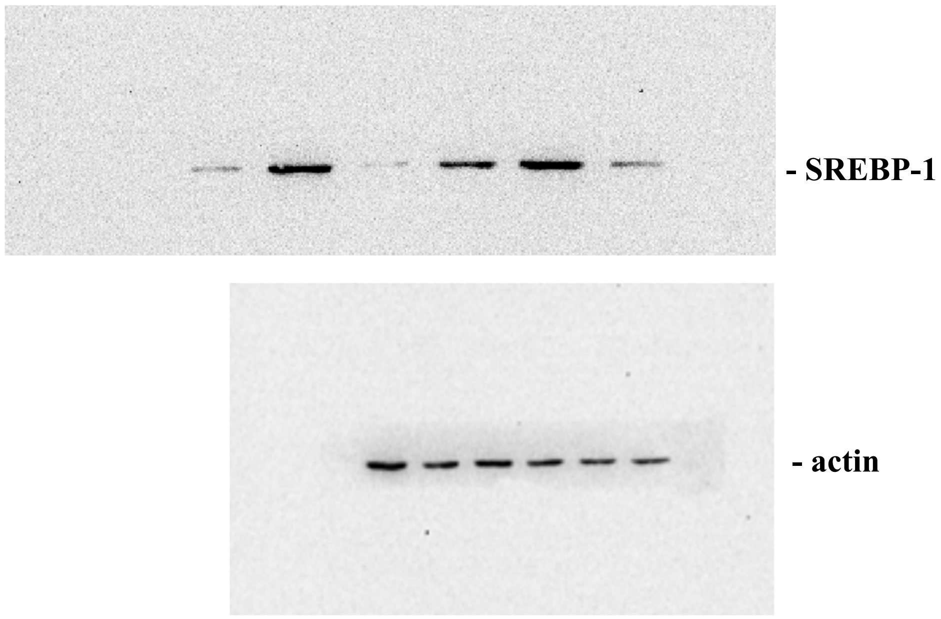

Figure 3D

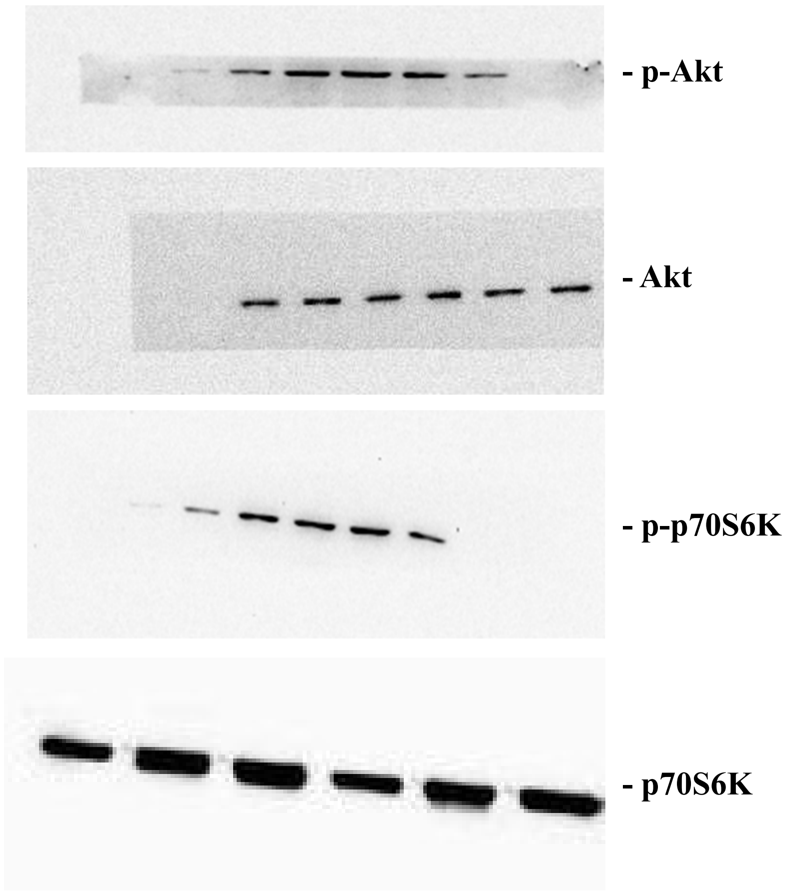

Figure S1: Gel images of Western blot in Figure 3.

Figure 5B

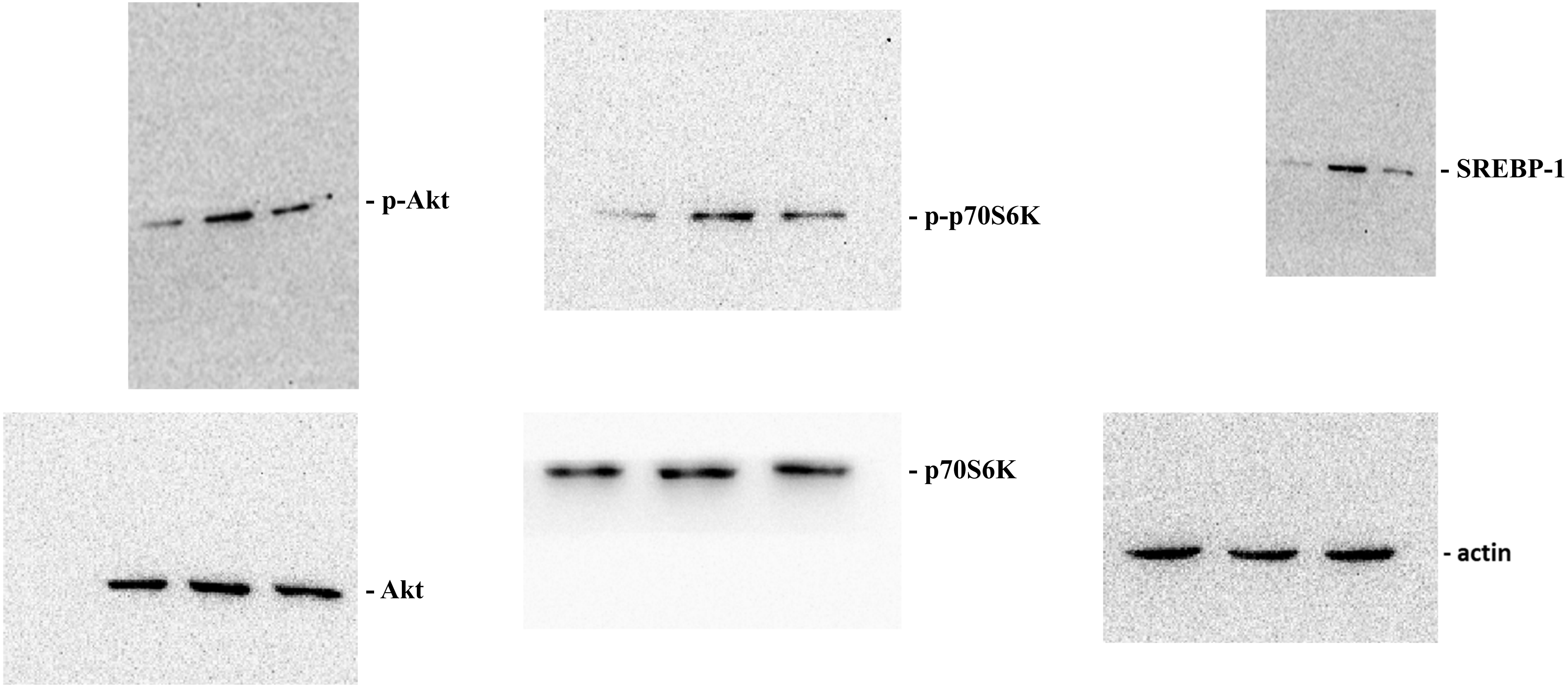

Figure S1: Gel images of Western blot in Figure 5.

Figure 6A

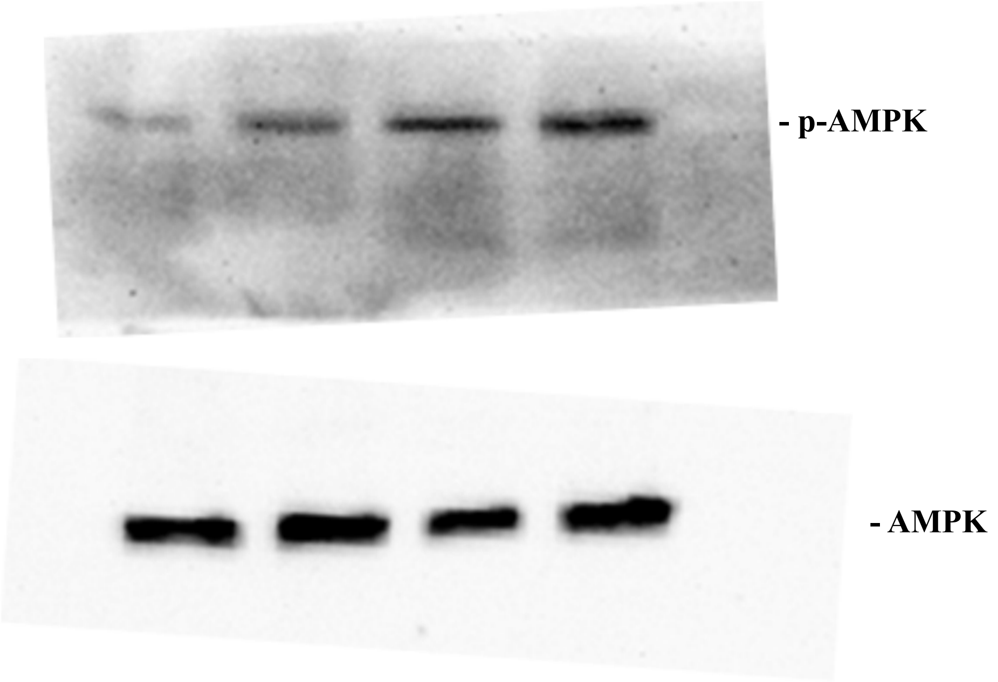

Figure S1: Gel images of Western blot in Figure 6.
